# New Potential Pharmacological Targets of Plant-Derived Hydroxyanthraquinones from *Rubia* spp.

**DOI:** 10.3390/molecules27103274

**Published:** 2022-05-19

**Authors:** Petko Alov, Merilin Al Sharif, Hristo Najdenski, Tania Pencheva, Ivanka Tsakovska, Maya Margaritova Zaharieva, Ilza Pajeva

**Affiliations:** 1 Institute of Biophysics and Biomedical Engineering, Bulgarian Academy of Sciences, 1113 Sofia, Bulgaria; petko@biophys.bas.bg (P.A.); merilin.al@biomed.bas.bg (M.A.S.); tania.pencheva@biomed.bas.bg (T.P.); itsakovska@biomed.bas.bg (I.T.); 2 The Stephan Angeloff Institute of Microbiology, Bulgarian Academy of Sciences, 1113 Sofia, Bulgaria; hnajdenski@gmail.com (H.N.); zaharieva26@yahoo.com (M.M.Z.)

**Keywords:** pseudopurpurin, DNA gyrase, DNA topoisomerase IV, antibacterial action, similarity-based screening, docking

## Abstract

The increased use of polyphenols nowadays poses the need for identification of their new pharmacological targets. Recently, structure similarity-based virtual screening of DrugBank outlined pseudopurpurin, a hydroxyanthraquinone from *Rubia cordifolia* spp., as similar to gatifloxacin, a synthetic antibacterial agent. This suggested the bacterial DNA gyrase and DNA topoisomerase IV as potential pharmacological targets of pseudopurpurin. In this study, estimation of structural similarity to referent antibacterial agents and molecular docking in the DNA gyrase and DNA topoisomerase IV complexes were performed for a homologous series of four hydroxyanthraquinones. Estimation of shape- and chemical feature-based similarity with (S)-gatifloxacin, a DNA gyrase inhibitor, and (S)-levofloxacin, a DNA topoisomerase IV inhibitor, outlined pseudopurpurin and munjistin as the most similar structures. The docking simulations supported the hypothesis for a plausible antibacterial activity of hydroxyanthraquinones. The predicted docking poses were grouped into 13 binding modes based on spatial similarities in the active site. The simultaneous presence of 1-OH and 3-COOH substituents in the anthraquinone scaffold were emphasized as relevant features for the binding modes’ variability and ability of the compounds to strongly bind in the DNA-enzyme complexes. The results reveal new potential pharmacological targets of the studied polyphenols and help in their prioritization as drug candidates and dietary supplements.

## 1. Introduction

The diverse pharmacological effects of plant polyphenols in parallel with their constantly increasing use in diets and as ethnomedicines justify the efforts for identification of the molecular mechanisms of their therapeutic action as well as finding their potential molecular targets. Recently, we have reported a study estimating structural similarity between plant-derived phenols and drug compounds from the DrugBank database [1] (https://www.drugbank.ca, last accessed on the 1 March 2022). A representative dataset of 75 phenols selected from the literature and a virtual library of more than 7000 drug compounds extracted from the DrugBank have been used [2]. Performing structural similarity-based virtual screening (OpenEye scientific software platform, https://www.eyesopen.com, last accessed on the 1 March 2022) of the DrugBank database, we have demonstrated that pseudopurpurin, a hydroxyanthraquinone from *Rubia* spp., was structurally similar to gatifloxacin, a synthetic broad-spectrum antibacterial agent.

Gatifloxacin is a representative of the fourth-generation fluoroquinolone family. It is known to work by inhibiting the bacterial enzymes DNA gyrase and DNA topoisomerase IV [3,4]. These enzymes have been considered attractive targets for discovery and design of biologically active compounds with antibacterial activity, due to their essential role for the maintenance of a proper DNA topology during transcription and replication [5]. In parallel, anthraquinones have been known as inhibitors of bacterial topoisomerases I and II [6], antibacterial effects [7], and biological activities against *Staphylococcus aureus* and *Bacillus subtilis* have been reported for *Rubia cordifolia* L. [8].

The structural similarity of pseudopurpurin to gatifloxacin pointed to both DNA-related enzymes as potential molecular targets of other hydroxyanthraquinones from *Rubia* spp. and motivated our further interest in investigating these natural compounds as potential inhibitors of the DNA gyrase and DNA topoisomerase IV enzymes.

In the present study, a homologous series of plant-derived hydroxyanthraquinones previously reported among the secondary metabolites typically found in *Rubia* spp. [9,10] was comparatively explored with regard to similarity with synthetic drugs shown to interact with DNA gyrase and DNA topoisomerase IV, namely, the synthetic fluoroquinolones (S)-gatifloxacin and (S)-levofloxacin. Furthermore, docking simulations of the compounds in the two bacterial enzymes were carried out to predict potential interactions and to map molecular modes of their possible antibacterial action. Overall, the carboxyl and hydroxyl groups in the structures of hydroxyanthraquinones were outlined as significant features predisposing to higher variability of the ligands’ behavior in the binding site, and stronger binding, thus suggesting the possible use of these hydroxyanthraquinones as appropriate lead structures.

## 2. Results

### 2.1. Evaluation of Structural Similarity

**Figure 1:**
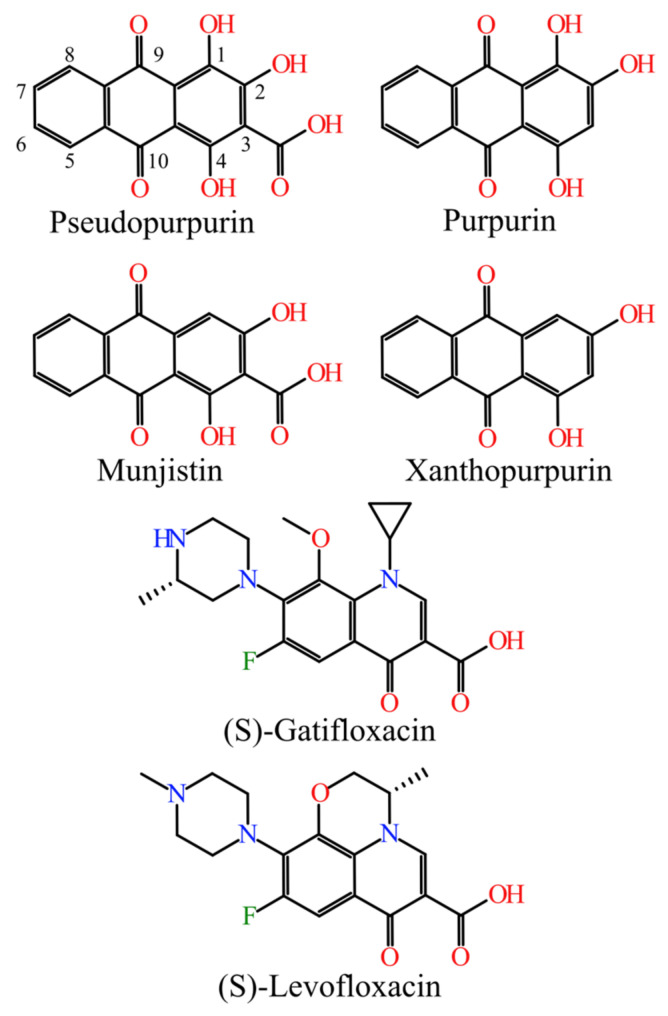
Chemical structures of the four naturally-occurring hydroxyanthraquinones and the two synthetic fluoroquinolones.

The chemical structures of the studied hydroxyanthraquinones (munjistin, pseudopurpurin, purpurin, and xanthopurpurin) and the two synthetic fluoroquinolone drugs from the DrugBank ((S)-gatifloxacin and (S)-levofloxacin) are shown in Figure 1.

The structural similarity of the studied anthraquinones to the fluoroquinolones was estimated considering that the shape and surface properties of the structures are crucial for the interactions of the bioactive compounds with the macromolecular targets. Thus, an overlay of the compared structures was performed using ROCS (see Section 4.2), trying to maximize both the shared volume (shape optimization of the overlay) and the alignment of functional groups with the same or similar properties (“color” optimization of the overlay). Two types of similarity scores were calculated—shape and “color”—as well as their sum (the so-called combo score).

The Tversky and Tanimoto scores produced similar results; thus, finally the Tanimoto scores were reported for estimation of similarity (Table 1). The shape-based similarity of the hydroxyanthraquinones to the two referent fluoroquinolones was higher compared to the one based on the chemical features. In both screenings based on either (S)-gatifloxacin or (S)-levofloxacin as a query (template) for shape-similarity evaluation, the scores pointed to pseudopurpurin as the most similar structure. Munjistin, however, was associated with the best alignment based on the chemical features’ similarity. The cumulative TanimotoCombo scores of pseudopurpurin and munjistin were very close (1.17 and 1.18 for (S)-gatifloxacin, and 1.20 and 1.23 for (S)-levofloxacin, respectively) indicating that the carboxylated hydroxyanthraquinones resemble more closely the structures of the referent fluoroquinolones.

### 2.2. Validation of the Docking Protocol for the Protein-DNA-Drug Complexes of DNA Gyrase and DNA Topoisomerase IV

**Figure 2:**
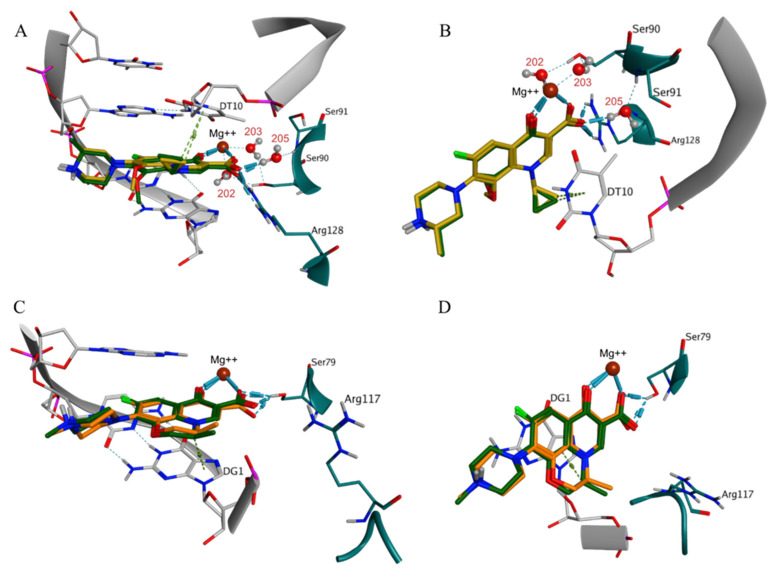
Redocking of the native ligands in the active sites of the protein-DNA-drug complexes presented in two different views: (**A**,**B**) DNA gyrase–(S)-gatifloxacin (X-ray pose in yellow, the best redocked pose in dark green); and (**C**,**D**) DNA topoisomerase IV–(S)-levofloxacin (X-ray pose in orange, the best redocked pose in dark green). The indices of the bridging water molecules HOH202, HOH203, and HOH205 are shown in red. The DNA molecules, including the nucleotides thymine 10 (DT10) and guanine 1 (DG1), are shown in grey; the proteins and their amino acid residues (Ser90, Ser91, Ser79, Arg128, and Arg117) are shown in teal; and the Mg++ ion is shown in brown. The intermolecular ionic and H-bond interactions are displayed with blue cylinders, while arene-H interactions—with light green cylinders.

The docking protocol was initially validated by redocking of the X-ray structures of the two antibacterial agents in their binding sites. The successful reproduction of the original X-ray poses of the two ligands within the top-scored docking poses was evidenced by the lowest RMSD values of 0.915 Å (for (S)-gatifloxacin) and 0.854 Å (for (S)-levofloxacin), corresponding to docking scores −22.3 kcal/mol and −19.4 kcal/mol, respectively. The characteristic ligand-receptor interaction patterns in the active sites of the two protein-DNA-drug complexes were successfully reproduced after redocking as well. In the case of (S)-gatifloxacin in DNA gyrase, H-bond interactions with the amino acid residues Ser91 and Ser90, and H-bond/ionic interactions with Arg128 were predicted in line with the originally observed ones for the 5BTF X-ray pose. In addition, the metal/ionic interactions with the Mg++ ion were reproduced (Figure 2A,B). The redocking in the binding site also reproduced contacts with three important bridging water molecules. The water molecules HOH202 and HOH203 together with the Mg++ ion participated in the formation of a complex H-bond network between the ligand and Ser90, while HOH205 interacted with the ligand and Ser91 only. Arene-H interaction with thymine 10 (DT10) was also reproduced in the simulation. Regarding (S)-levofloxacin in DNA topoisomerase IV, the successfully reproduced interactions were related to direct H-bond with Ser79 and metal/ion interactions with the Mg++ ion (Figure 2C,D).

### 2.3. Molecular Docking of Hydroxyanthraquinones

The molecular docking simulations of the hydroxyanthraquinones in the DNA gyrase complex produced 97 poses with scores between −44.7 and −9.4 kcal/mol, while in the DNA topoisomerase IV complex, the predicted poses were 98 and the respective scoring range was between −36.2 and −7.3 kcal/mol. The scores of the redocked native ligands, −22.3 kcal/mol ((S)-gatifloxacin) and −19.4 kcal/mol ((S)-levofloxacin), were used as cutoff values for filtering the hydroxyanthraquinones’ docking poses. This step resulted in two sets of predicted docking poses with binding energies comparable and stronger than that of the reference fluoroquinolones. The filtered poses in DNA gyrase were 52 with docking scores between −44.7 and −22.6 kcal/mol and those in DNA topoisomerase IV were 38 with scores between −36.2 and −19.5 kcal/mol. These poses were visually analyzed and classified into 13 groups (designated here as clusters of binding modes, BMs), each with a different spatial orientation in the active sites (Table S1).

**Figure 3:**
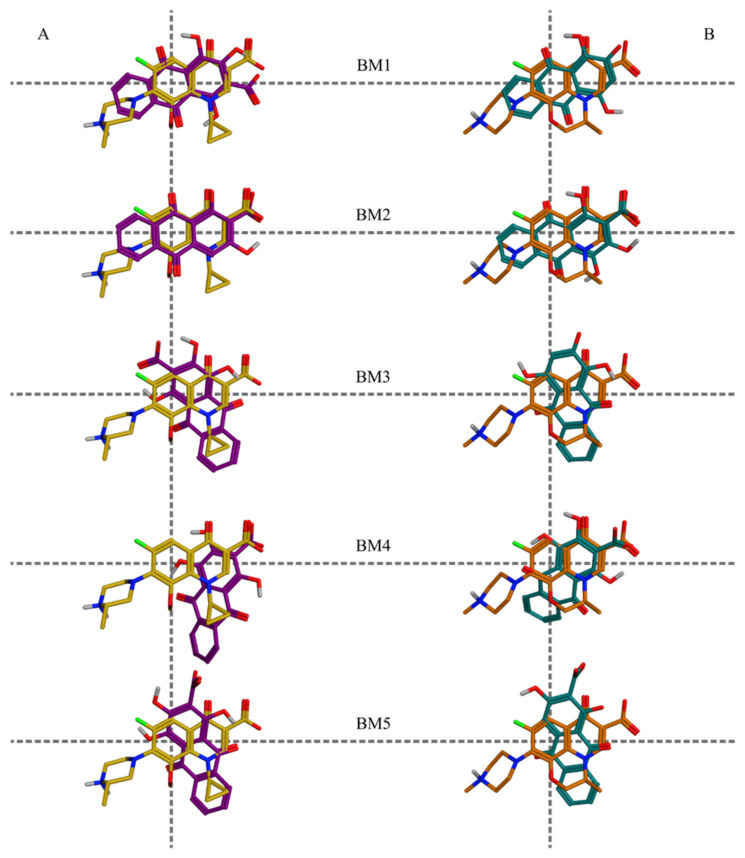
Shared binding modes predicted in both enzymes—DNA gyrase and DNA topoisomerase IV: (**A**) overlay on (S)-gatifloxacin (yellow) of the following hydroxyanthraquinones (dark violet), pseudopurpurin (BM1, BM3, BM4, and BM5) and munjistin (BM2); (**B**) overlay on (S)-levofloxacin (orange) of the following hydroxyanthraquinones (teal), purpurin (BM1 and BM3), pseudopurpurin (BM2 and BM4), and munjistin (BM5).

**Figure 4:**
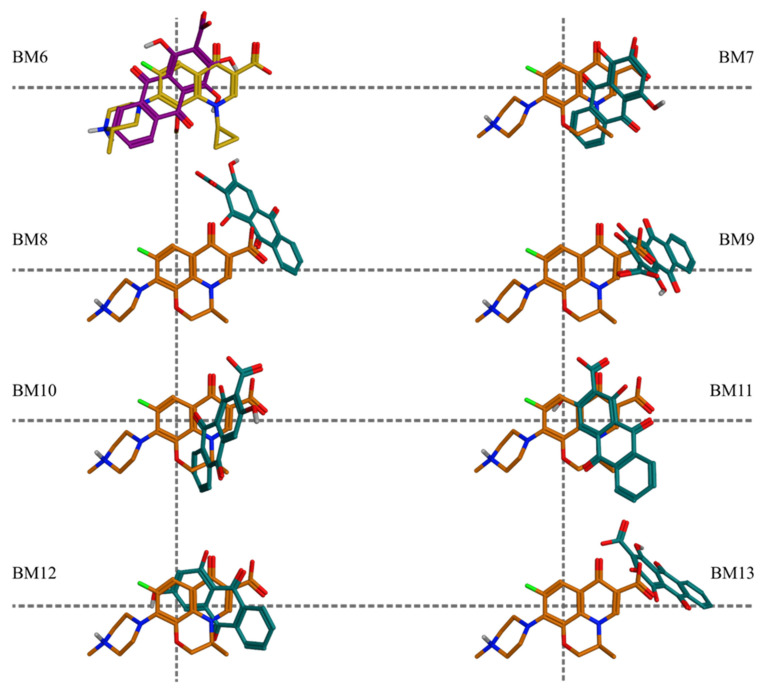
Unique binding modes predicted in the two enzymes. In DNA gyrase, BM6 is illustrated by pseudopurpurin (dark violet), overlaid on (S)-gatifloxacin (yellow); in DNA topoisomerase IV, the BMs are illustrated by overlay on (S)-levofloxacin (orange) of the following hydroxyanthraquinones (teal): purpurin (BM7), munjistin (BM8, BM10, and BM11), pseudopurpurin (BM9 and BM13), and xanthopurpurin (BM12).

When analyzing the poses in the binding sites, three main topological features were taken into account for the BMs clustering: (i) the relative orientation of the plane of the hydroxyanthraquinone scaffold compared to that of the referent fluoroquinolones; (ii) the overlay of the ring system of the hydroxyanthraquinone scaffold with respect to the rings of the referent fluoroquinolones; and (iii) location of the aromatic C-atom in position 3 of the hydroxyanthraquinone scaffold (Figure 1) with respect to the horizontal or the vertical axes of rotation of the predicted alternative (flipped) binding modes (Table S1). The docking poses were analyzed by comparing both the poses of the distinct ligand structures within a single protein simulation and the two output databases of the structures docked into the active sites of the two targets. The frequency of a given BM within the particular simulation group (DNA gyrase, DNA topoisomerase IV, or both taken together) was calculated with respect to the total number of filtered poses in the corresponding group. Among the 13 BMs, 6 were associated with poses in DNA gyrase and 12 in DNA topoisomerase IV complexes; of them, 5 BMs appeared in the docking outputs from both simulation groups (shared BMs, Table S1, Figure 3). One BM was unique for the DNA gyrase-based docking and 7—for the DNA topoisomerase IV-based docking outputs (unique BMs, Table S1, Figure 4). Thus, in the distribution of the poses by binding modes, the shared poses for the simulations in the two complexes were 38% of the whole array of poses, the poses unique to the DNA gyrase-based simulation constituted 8% of all poses, and those predicted as unique for the DNA topoisomerase IV complex were 54% of all poses.

The docking poses were further filtered taking into account the ionization state of the hydroxyanthraquinones. The 3-COOH groups in pseudopurpurin and munjistin were predicted to be deprotonated at physiological pH in the calculations using all three algorithms (Table S2). The protonated (neutral) and deprotonated 2-OH group of purpurin and xanthopurpurin appeared in different ratios in the predictions; thus, both ionization states of these compounds were considered. These states of hydroxyanthraquinones are designated below as highly probable ones, while the rest were considered as ionization states of lower probability.

The statistics of the unique and shared BMs was different depending on the ionization states considered. The hydroxyanthraquinones in the highly probable ionization states were predicted to have 28 poses in DNA gyrase (−31.0 to −22.6 kcal/mol) and 13 in poses in DNA topoisomerase (−24.1 to −19.5 kcal/mol). This subset included states with deprotonated 3-COOH group for pseudopurpurin and munjistin and deprotonated 2-OH group for purpurin and xanthopurpurin. Although neutral states were also assigned to these compounds, only one pose in a neutral state was present (that of purpurin from DNA gyrase-based simulation left after the initial filtering by docking score thresholds). The poses in this subset displayed six different BMs: three shared, two unique for the DNA gyrase complex, and one unique for the DNA topoisomerase IV complex (Table 2).

In the DNA gyrase-based simulation pseudopurpurin, munjistin and purpurin in their highly probable ionization states were presented by three poses each within BM1, while only one pose of xanthopurpurin appeared in BM1. The BM3 was mainly associated with purpurin (four poses), followed by pseudopurpurin (two poses) and a single pose of xanthopurpurin. Two poses of pseudopurpurin were included in BM4, together with single poses of munjistin and purpurin. Purpurin was the only hydroxyanthraquinone, the poses of which did not appear in BM6. Pseudopurpurin (two poses) and munjistin (one pose) were classified also to BM5.

Regarding the shared BM1, BM3, and BM4, the poses in DNA topoisomerase IV were distributed in slightly different proportions compared to DNA gyrase. In BM1, munjistin and xanthopurpurin were equally presented (by two poses each), purpurin appeared with one pose while pseudopurpurin was missing. In BM3 purpurin (three poses) and xanthopurpurin (one pose) were present, and only two poses of pseudopurpurin were classified in BM4. In the subset of poses of highly probable ionization states, BM2 (presented by two pseudopurpurin’s poses) was unique to the DNA topoisomerase IV-based simulation.

In the subset of poses in ionization states of lower probability 24 poses were predicted in DNA gyrase (−44.6 to −24.3 kcal/mol), and 25 poses in DNA topoisomerase IV (−36.2 to −20.6 kcal/mol). For this subset, BM1, BM2, BM3, and BM5 were shared by the hydroxyantraquinones’ poses in both targets. The poses in DNA topoisomerase IV were associated with variety of BMs (BM7, BM8, BM9, BM10, BM11, BM12, and BM13), unique for this target, compared to only two unique BMs (BM4 and BM6) for the DNA gyrase-based simulation. The poses in this subset were estimated to have better (more negative) docking scores, compared to those in the highly probable ionization states. This could be explained by the increased number of deprotonated 1,2,4-OH substituents of the hydroxyanthraquinone scaffold.

## 3. Discussion

DNA gyrase and DNA topoisomerase IV have been classified as type IIA topoisomerases. Their mechanism of action involves formation of a transient covalent bond to the 5ʹDNA-phosphate of both strands of the DNA duplex and to function via a strand-passage mechanism [11]. The inhibitory effect of quinolones has been associated with binding to the enzyme-DNA complexes, thus trapping the enzyme targets on bacterial DNA as ternary drug-enzyme-DNA complexes [12].

In the current study, munjistin, purpurin, and xanthopurpurin were selected based on the common structural scaffold they share with the previously highlighted hit compound pseudopurpurin [2]. The scores of structural similarity bewteen the four investigated hydroxyanthraquinones to the referent fluoroquinolones were quite close, those of the carboxylated pseudopurpurin and munjistin being slightly higher than the ones of the non-carboxylated purpurin and xanthopurpurin. Similar trends were outlined also for (S)-levofloxacin. Overall, slightly higher similarity was reported to (S)-levofloxacin compared to (S)-gatifloxacin.

The molecular docking simulations of the structures with the highly probable ionization states in the protein-DNA-drug complexes of DNA gyrase and DNA topoisomerase IV supported the initial hypothesis about the potential mechanism(s) of the antibacterial effects of *Rubia* spp. Pseudopurpurin and purpurin appeared most frequently among the best scored docking poses. In particular, for both targets pseudopurpurin appeared above the median of the docking scores and within the best-scored quartiles (the 75-th percentile) in all ionization states, while purpurin appeared among the top-ranked poses representative for the highly probable ionization states, underlining the relevance of the 1-OH substituent for the affinities to the targets. The most negatively scored BMs were BM1, BM2, BM3, and BM4, respectively. According to the results, pseudopurpurin and munjistin have the highest number of different BMs in DNA gyrase suggesting higher probability of binding to this enzyme.

The variability of the molecular features of the four hydroxyanthraquinones, allowed for a deeper analysis of the relevance of the 3-COOH and the 1-OH substituents to the molecular modes of action of pseudopurpurin (Figure 1). This was clearly outlined in the detailed PLIF analysis of the binding modes in the active sites of the two DNA-enzyme complexes and the ranking of the binding modes by their average docking scores (Tables S3 and S4). Such complementary approach combining knowledge for the chemical domain of the studied series of compounds, the predicted patterns of ligand-receptor interactions, and the quantitative approximations of ligands’ affinities has been successfully applied in our previous investigations focused on prediction and classification of potential molecular modes of action of multiple Phase I metabolites of a triterpenoid [13].

The detailed comparative analysis of the ligand-receptor interaction fingerprints within the subset of poses in highly probable ionization states revealed important trends regarding the structural variability of the studied hydroxyantraquinones. The ranking by the docking scores clearly distinguished affinity-related interaction patterns correlating the top scoring poses to frequently occurring interactions between the Mg++ ion and the 1,2-OH substituents simultaneously. The bottom-scored pattern in most cases involved simultaneous interactions of the 2-OH and the 3-COOH substituents with the Mg++ ion, together with direct interactions of the 3-COOH group with Arg128 or water-bridged ones with Ser90 and Ser91 (Table S3).

**Figure 5:**
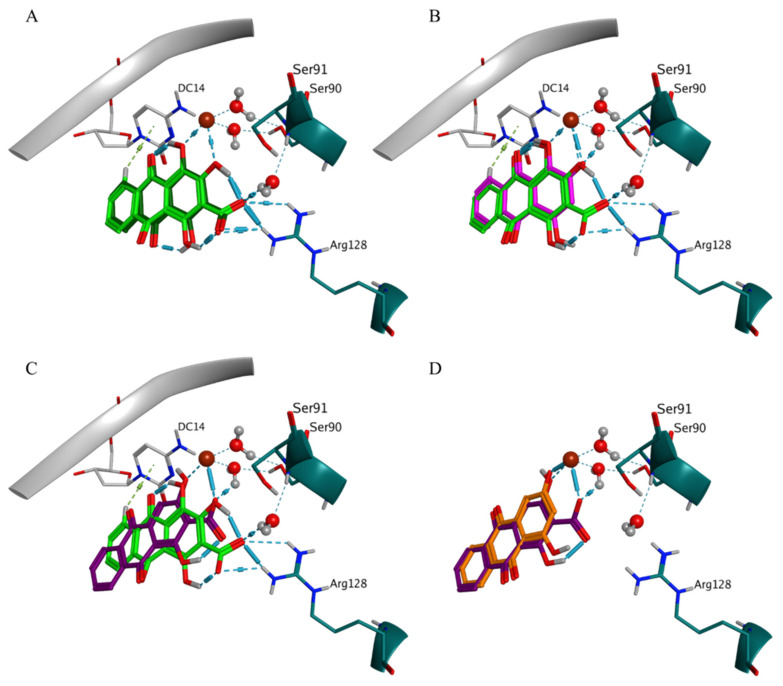
Binding mode 1 in the DNA gyrase complex. (**A**) comparison of the ligand-receptor interactions of two poses of pseudopurpurin (dark and light green), DC14—cytosine 14; and (**B**) comparison of the ligand-receptor interactions of purpurin (magenta) to those of two pseudopurpurin‘s poses (green). Comparison of the ligand-receptor interactions of munjistin (dark violet) to those of: (**C**) pseudopurpurin (green) and (**D**) xanthopurpurin (orange). Mg++ is shown in brown and bridging waters are shown in atom color coding in sticks and balls rendering.

Pseudopurpurin and its non-carboxylated analogue purpurin shared three similar BMs in DNA gyrase, i.e., BM1, BM3, and BM4, and were the compounds with the most negative best and average scores (Figure 5). Possessing the higher number of substituents, pseudopurpurin also displayed the most diverse ligand-receptor interaction patterns characterized by the highest number of key partnering residues (an amino acid residue, a nucleotide, a Mg++ ion or a water molecule) (Table S3). Compared to its analogue lacking 1-OH substituent (munjistin), pseudopurpurin performed a higher number of interactions with Arg128, a significantly higher number of water-mediated (HOH205) H-bond interactions with Ser91, and interactions with nucleotide and with a phosphorylated tyrosine 129 (Ptr129) (Table S3). In one of the BM1 poses pseudopurpurin performed arene-H interaction with DC14 through the distal anthraquinone ring (Figure 5A). This interaction was predicted neither for purpurin (Figure 5B), nor for munjistin (Figure 5C) and xanthopurpurin (Figure 5D). The lack of interactions with Arg128 in BM1 of munjistin, compared to pseudopurpurin was associated with the engagement of the 3-COOH substituent with the coordination of Mg++, while this was performed by 1-OH and/or 2-OH substituents in the case of pseudopurpurin (Figure 5C). Due to the lack of 3-COOH substituent, purpurin and xanthopurpurin did not perform interactions with Arg128 (Figure 5B,D).

**Figure 6:**
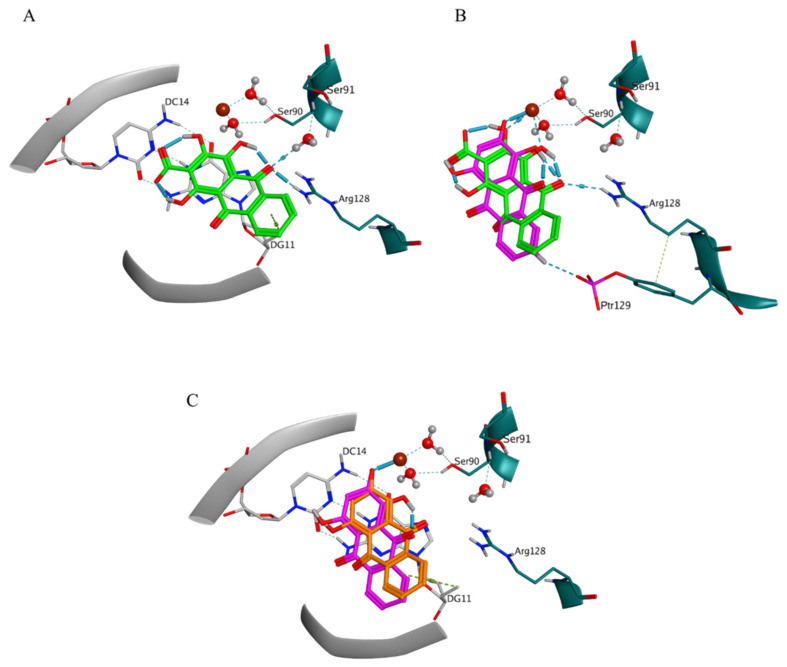
Binding mode 3 in the DNA gyrase complex. (**A**) ligand-receptor interactions of one of the two poses of pseudopurpurin in BM3, DC14—cytosine 14, DG11—guanine 11; (**B**) comparison of the ligand-receptor interactions of purpurin (BM3, magenta) to those of the second pose of pseudopurpurin in BM3 (green), Ptr129—phosphorylated tyrosine 129; and (**C**) comparison of the ligand-receptor interactions of purpurin (BM3, magenta) to that of xanthopurpurin (BM3, orange).

The interactions with DG11 and Ptr129 were rarely predicted (Table S3). In BM3, DG11 appeared in the ligand-receptor interaction patterns of pseudopurpurin (Figure 6A) and xanthopurpurin (Figure 6C), and purpurin displayed H-bond interaction with Ptr129 (Figure 6B). The ligand-receptor interaction pattern of the two pseudopurpurin’s poses grouped in BM3 involved an obligatory interaction between the carbonyl substituent at position 9 and Arg128 (Figure 6A,B). The two poses, however, displayed either interaction of the carbonyl group at position 9 with the HOH205-Ser91, combined with interaction of the distal anthraquinone ring and guanine 11 (DG11) (Figure 6A), or interactions between the Mg++ ion and the 1,2-OH substituents (Figure 6B).

The binding patterns of purpurin and xanthopurpurin were lacking the interactions with Arg128 and HOH205-Ser91, predicted for their carboxylated analogues (Table S3).

The deeper analysis of the participation of the different substituents in the performed ligand-receptor interactions explained some of the differences in their binding patterns. The lack of a 3-COOH substituent in purpurin resulted in a higher frequency of simultaneous involvement of the 1-OH and 2-OH substituents in the coordination of the Mg++ ion compared to pseudopurpurin. The metal/ion interaction pattern of the latter engaged with an equal frequency the simultaneous participation of the 2-OH and 3-COOH substituents on the one hand, and of the 1-OH and 2-OH substituents, on the other. Logically, xanthopurpurin relied only on the 2-OH substituent for this interaction due to lack of both 1-OH and 3-COOH substituents. The lack of a 3-COOH substituent affected the binding behavior of purpurin in BM1 and BM4 regarding the water-bridged interaction with Ser90. Purpurin performed H-bond interaction through its 2-OH substituent, instead of involving a 3-COOH substituent as predicted for munjistin and pseudopurpurin in these BMs (Table S3).

**Figure 7:**
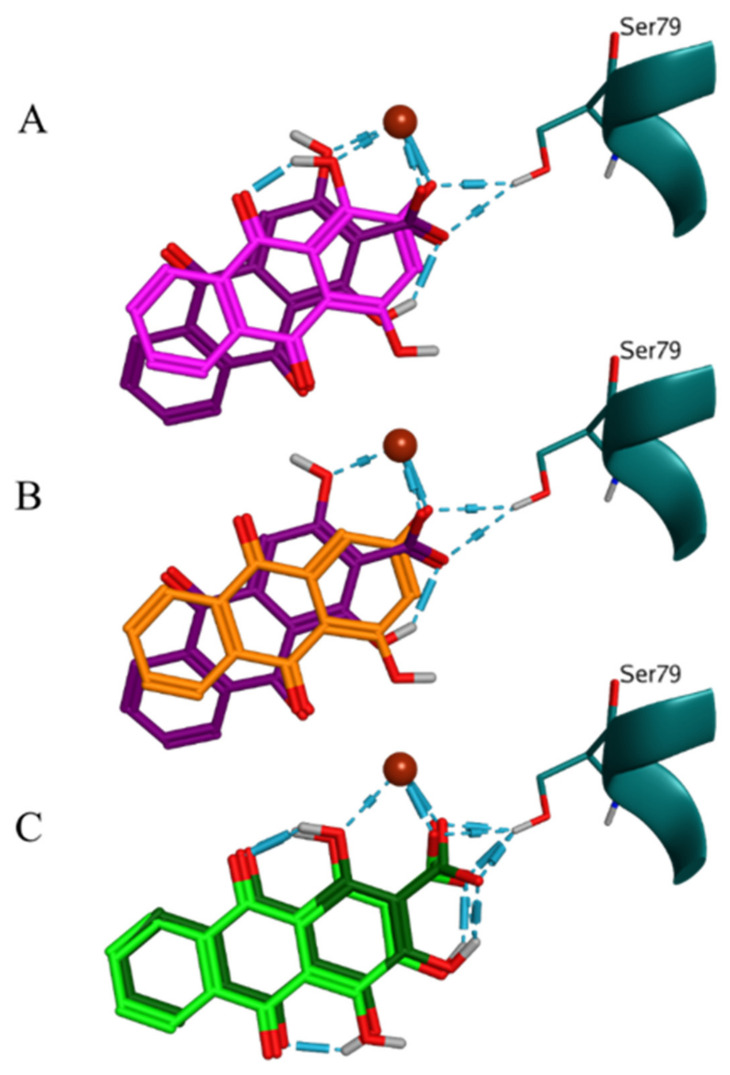
Binding modes in the DNA topoisomerase IV complex. (**A**) comparison between the ligand-receptor interactions of munjistin (BM1, violet) and purpurin (BM1, magenta); (**B**) comparison between the ligand-receptor interactions of munjistin (BM1, violet) and xanthopurpurin (BM1, orange); and (**C**) comparison between two poses of pseudopurpurin in BM2.

The ligand-receptor interaction patterns in DNA topoisomerase IV were characterized by metal/ion interactions for all hydroxyanthraquinones similarly to the patterns in DNA gyrase (Table S4). Due to the intrinsic features of the complex in this simulation, the predicted interactions with Ser79 were direct (Figure 7). The poses differed mostly in the type of the substituents involved in the metal/ion interaction with the Mg++ ion and the H-bond interaction with Ser79. Pseudopurpurin and munjistin interacted with the Mg++ ion exclusively by the 3-COOH substituent alone or in combination with the 2-OH substituent (Figure 7). In particular, pseudopurpurin performed this interaction also in combination with the 4-OH substituent. Compared to the DNA gyrase docking output in this complex the total number of poses and the variety of binding modes was significantly lower (Table 2). Nevertheless, pseudopurpurin was still with the highest number of poses. Munjistin, however, was presented only by two poses, both in the shared for the two targets BM1 (Figure 7A). All hydroxyanthraquinones, except pseudopurpurin, were shown to display this BM. In DNA topoisomerase IV, the interactions of purpurin and xanthopurpurin with Ser79 in BM1 were performed through 1-OH and 2-OH for purpurin (Figure 7A) and 2-OH for xanthopurpurin (Figure 7B), respectively, while munjistin involved a combination of its 2-OH and 3-COOH substituents instead (Figure 7A,B). Pseudopurpurin was the only hydroxyanthraquinone with poses classified in the BM2 group. This binding mode represents a variation of BM1 (Figure 5A), which was vertically flipped around the imaginary horizontal axis of the three-ring hydroxyanthraquinone scaffold (Table S1). The two pseudopurpurin’s poses included in BM2 had almost similar ligand-receptor interaction patterns. The only difference between them was the appearance of one more metal/ion interaction between the 4-OH substituent and the Mg++ ion (Table S4, Figure 7C). Similarly, to the scoring in the DNA gyrase-based simulation, the best docking scores of the two 1-OH substituted hydroxyanthraquinones were the most negative ones among the best scores of all analogues. This was also valid for their average scores (Table 2).

The analysis of the frequencies of participation of each individual substituent in ligand-receptor interactions (Table S5) outlined the central place of the 3-COOH substituent in the receptor-binding patterns of the top scored poses of pseudopurpurin. In addition, the compensatory role of the 4-OH substituent in the interactions of the analogue which lacks the 1-OH substituent (munjistin) was evident. This is related to BMs, which less frequently involve the 3-COOH compared to the interaction pattern of the 1-OH containing analogue (pseudopurpurin). Such a compensatory role in the case of pseudopurpurin’s non-carboxylated analogue purpurin was predicted for the 2-OH substituent. It could be concluded that the absence of a 1-OH substituent results in a spatial shift of the poses to favor a less frequent involvement of the 3-COOH substituent; the absence of the 3-COOH substituent favors the more frequent involvement of the 2-OH substituent. The median values of the hydroxyanthraquinones’ docking scores for DNA topoisomerase IV were comparable. However, in the DNA gyrase-based simulations the presence of the 1-OH substituent is related with better docking scores as evident from the median values of the poses, while the presence of the 3-COOH substituent increases the total number of the predicted poses and the number of ligand-receptor interactions. All these observations underline the role of the 1-OH and 3-COOH substituents for the ligand interactions and point to pseudopurpurin as the most promising molecular scaffold in the series.

Based on these results, further studies could be designed to explore more comprehensively the mechanistically relevant binding behavior of this class of naturally-occurring compounds in the two target complexes. Given the fact that these hydroxyantraquinones have been known as constituents of orally applied traditional medicinal preparations [14], and even commonly consumed beverages in some regions [15,16], an in silico investigation of the potential metabolites of these compounds might be performed as a next step in this modelling workflow. Another intriguing aspect that deserves further attention is the influence of the protonation states of the ligands and their targets on the ligand-receptor interactions upon different pH-related settings. This is especially interesting in relation to the pathological action of *M. tuberculosis* and is in line with the therapeutic paradigm exploiting pH-dependent phenomena of relevance to this pathogen [17,18].

## 4. Data and Methods

### 4.1. Structural Data

The structural formulas of the investigated compounds are shown in Figure 1. Four hydroxyanthraquinones constituted the homologous series studied. They differ by the presence/absence of a carboxyl group (-COOH) in position 3 and by the presence/absence of hydroxyl substituents (-OH) in positions 1 and 2: pseudopurpurin (1,2,4-OH and 3-COOH), purpurin (1,2,4-OH), munjistin (2,4-OH and 3-COOH), and xanthopurpurin (2,4-OH). The compounds were assessed for their structural similarity to the synthetic ligands of DNA gyrase and DNA topoisomerase IV– (S)-gatifloxacin and (S)-levofloxacin.

The enzyme structures were retrieved from the Protein Data Bank [19] (PDB, https://www.rcsb.org, last accessed on the 1 March 2022). The X-ray complexes of DNA gyrase–DNA–(S)-gatifloxacin (PDB ID 5BTF, *Mycobacterium tuberculosis*) and DNA topoisomerase IV–DNA–(S)-levofloxacin (PDB ID 3RAE, *Streptococcus pneumoniae*) were selected, taking into account the ligands’ identity/similarity to the previously highlighted drug hit (gatifloxacin) and the relatively good crystallographic resolutions (2.61 and 2.90 Å for 5BTF and 3RAE, respectively).

### 4.2. Similarity-Based Virtual Screening

Generation of molecular conformations for the neutral states of the compounds and similarity evaluation were performed using the OpenEye Scientific software platform. Multi-conformer structural databases were created using OMEGA 4.1.2.0 [20]; shape- and chemical feature-based overlays of the conformers were performed with ROCS 3.4.3.0 [21]. The compounds’ similarity was evaluated using Tanimoto and Tversky scores, focused on shape (ShapeTanimoto, FitTversky, and RefTversky) or on chemical features’ (ColorTanimoto, FitColorTversky, and RefColorTversky) similarity. The cumulative scores (TanimotoCombo, FitTverskyCombo, and RefTverskyCombo) were calculated as a sum of the respective shape and chemical features scores.

### 4.3. Molecular Docking

MOE software platform (Molecular Operating Environment 2020.09, https://www.chemcomp.com, last accessed on the 1 March 2022) was used. The database with the 3D structures of the ligands was created using the MOE Molecule Builder tool based on the SMILES codes of the compounds as retrieved from the NIH PubChem system (https://pubchem.ncbi.nlm.nih.gov, last accessed on the 1 March 2022). The ionization states of the structures at a physiological pH 7.4 were assessed using the software platforms ACD/Percepta 2021.2.0 (https://www.acdlabs.com, last accessed on the 1 March 2022; ACD/pKa GALAS module) and ChemAxon 14.8.25.0 (https://chemaxon.com, last accessed on the 1 March 2022) protonation plugin (mode—macro, T 298K, min basic pKa –2, max acidic pKa 16, consider tautomerization/resonance) and the MOE Molecule Wash tool (protonation mode set to “Dominant”) (Table S2). Further steps of the molecular database preparation were performed in MOE, including partial charges calculation and energy optimization of the structures. The PDB complexes were prepared using the MOE 3D Protonation tool with the following settings: T 310K, pH 7.4, and ion concentration 0.152 mol/L.

Molecular docking was performed using the default Amber10 EHT force field in a rigid receptor/flexible ligand mode, considering both the receptor and the solvent. The Mg++ ion and the water molecules available in the binding sites were kept during the simulations. The binding energies of the compounds in the active sites of the enzymes were approximated by the docking scores calculated with the London dG scoring function (the more negative docking scores correspond to better interactions). The same function was applied both at the placement stage (using a triangle matching method) and at the refinement stage with a setting for up to 30 output poses per ligand at each stage. The MOE PLIF tool (Protein–Ligand Interaction Fingerprints) was used for analysis of the interactions patterns in the binding sites. Interactions such as hydrogen bonds, metal/ionic interactions, and arene attraction were further analyzed according to the participating residues of the complexes and the hydroxyanthraquinone substituents involved.

## 5. Conclusions

In this study, we combined several in silico methods in a multistep sequential manner—shape and chemical features’ similarity evaluation, structure-based molecular docking, and analysis of the protein-ligand interactions—to comprehensively estimate the potential of selected polyphenols from the chemical class of hydroxyanthraquinones to interact with the bacterial enzymes DNA gyrase and DNA topoisomerase IV. At each of these steps the results were encouraging and supportive for the correctness of our approach proven to be successful in a recent study of structurally more complex polyphenols [22].

Four hydroxyanthraquinones—pseudopurpurin, purpurin, munjistin, and xanthopurpurin—naturally occurring in *Rubia* spp. and varying by the substituents at positions 1, 2, and 3 were thoroughly investigated to reveal the relevance of their structural features for the interactions with the enzymes.

Pseudopurpurin and munjistin were found to be more structurally similar to the reference fluoroquinolones (S)-gatifloxacin and (S)-levofloxacin than their non-carboxylated analogues. Docking poses of pseudopurpurin and munjistin in the active sites of the protein-DNA complexes of DNA gyrase and DNA topoisomerase IV revealed their plausible molecular modes of action against Gram-positive and Gram-negative bacteria. The most populated binding modes reproduced the ligand-receptor interaction patterns of the X-ray poses of the referent fluoroquinolones and displayed additional specific interactions with key residues in the binding sites of the complexes. The anthraquinones’ docking scores were better than those of the referent fluoroquinolones, suggesting the possibility for stronger binding of the studied polyphenols to the DNA gyrase and DNA topoisomerase IV protein-DNA complexes compared to their co-crystalized ligands. The binding behavior of pseudopurpurin and munjistin was characterized by a wider range of diverse binding modes, which could be attributed mainly to the contribution of the 1-OH and 3-COOH substituents. This was also evident from the detailed ligand-receptor interaction analysis, which revealed that the presence of both substituents reflects on the ability of the ligands to interact with more key residues in the pocket. A similar trend was observed in the context of predicted ligand’s affinity to the binding sites with emphasis on the 1-OH substituent, present in purpurin and pseudopurpurin.

The results outline the studied hydroxyanthraquinones, and in particular pseudopurpurin, as promising lead structures for further drug development of antibacterial agents with potential to interact with the DNA gyrase and DNA topoisomerase IV. Design of appropriate experimental in vitro studies is ongoing to confirm the relevance of the in silico predictions.

The combined in silico approach used in this study could be applied to any other bioactive compounds of natural origin which putative molecular targets and mechanisms of action are still unknown or need to be elucidated at a molecular level.

## Figures and Tables

**Table 1:** Evaluation of shape- and chemical feature (“color”)-based similarity between the referent fluoroquinolones ((S)-gatifloxacin and (S)-levofloxacin), and the hydroxyanthraquinones—munjistin (M), pseudopurpurin (PP), purpurin (P), and xanthopurpurin (X). The highest similarity scores for shape, chemical features, and cumulative are bolded.

Query Fluoroquinolone	(S)-Gatifloxacin				(S)-Levofloxacin			
Hydroxy- Anthraquinone	M	PP	P	X	M	PP	P	X
ShapeTanimoto	0.72	**0.76**	0.75	0.73	0.76	**0.79**	0.77	0.75
ColorTanimoto	**0.46**	0.41	0.26	0.22	**0.47**	0.41	0.26	0.23
TanimotoCombo	**1.18**	1.17	1.02	0.95	**1.23**	1.20	1.03	0.97

**Table 2:** Summary of the predicted binding modes and docking scores (best, average, and median) of the studied hydroxyanthraquinones (in ionization states of higher probability) in the docking simulations in DNA gyrase and DNA topoisomerase IV. The BMs shared between the docking outputs of the two targets are bolded.

**Enzyme**	**DNA Gyrase**		**DNA Topoisomerase IV**	
**Compound** **(Substituents)**	**Binding Modes, No.**	**Best/** **Average/Median** **Docking Score, kcal/mol**	**Binding Modes, No.**	**Best/** **Average/Median** **Docking Score, kcal/mol**
**Pseudopurpurin** **(1,2,4-OH; 3-COOH)**	**1, 3, 4**, 5, 6	−28.7/−25.5/−25.1	2, **4**	−23.2/−22.2/−22.1
**Munjistin** **(2,4-OH; 3-COOH)**	**1, 4**, 5, 6	−25.5/−24.2/−24.1	**1**	−22.1/−21.7/−21.7
**Purpurin** **(1,2,4-OH)**	**1, 3, 4**	−31.0/−27.1/−26.7	**1, 3**	−24.1/−21.9/−21.9
**Xanthopurpurin** **(2,4-OH)**	**1, 3**, 6	−25.9/−24.7/−24.9	**1, 3**	−22.2/−21.5/−22.2

## Data Availability

Data is contained within the article or Supplementary Materials.

## Supplementary Materials

The following supporting information can be downloaded at https://www.mdpi.com/article/10.3390/molecules27103274/s1. Table S1: Distribution of the poses among the different binding modes in DNA gyrase and DNA topoisomerase IV and description of the spatial arrangement of the binding modes (BMs). Table S2: Predicted ionization states of the structures and their abundance at pH 7.4. Table S3: Binding modes in DNA gyrase: docking scores, ligand-receptor interaction fingerprints with respect to the hydroxyanthraquinone scaffold and the key entities in the complex, and a detailed ligand-receptor interaction pattern. Table S4: Binding modes in DNA topoisomerase IV: docking scores, ligand-receptor interaction fingerprints with respect to the hydroxyanthraquinone scaffold and the key entities in the complex, and a detailed ligand-receptor interaction pattern. Table S5: Frequency of involvement in ligand-receptor interactions of the top-scored poses based on the median value of the docking scores for each hydroxyanthraquinone in DNA gyrase and DNA topoisomerase IV.
