# Supplementary material for: New Potential Pharmacological Targets of Plant-Derived Hydroxyanthraquinones from Rubia spp
Source: Molecules. 2022 May 19;27(10):3274. doi: 10.3390/molecules27103274 (PMC9145346; doi:10.3390/molecules27103274)
Supplement: Supplementary file 1 [file molecules-27-03274-s001.zip › molecules-1703435-supplementary/molecules-1703435-supplementary_round1_revision/Table_S5.pdf]

Supplementary Material

Table S5. Frequency of involvement in ligand-receptor interactions of the top-scored poses based on the median value of the docking scores for each hydroxyanthraquinone in DNA gyrase and DNA topoisomerase IV. The substituents appearing most frequently in the ligand-receptor interaction pattern of each hydroxyanthraquinone is shown in bold.

**Receptor**	**Compound**	**Score (median, kcal/mol)**	**Best poses / all poses (number)**	**1-OH**	**2-OH**	**3-COOH**	**4-OH**	**9=O**
**DNA Gyrase**	Pseudopurpurin	–25.12	5 / 10	2	2	**5**		3
	Munjistin	–24.12	4 / 7			3	**4**	
	Purpurin	–26.70	3 / 8	2	**4**			
	Xanthopurpurin	–24.87	2 / 3		2			
**DNA Topoisomerase IV**	Pseudopurpurin	–22.07	1 / 4			**2**		
	Munjistin	–21.74	1 / 2		1	**2**		
	Purpurin	–21.89	2 / 4	2	**3**			
	Xanthopurpurin	–22.18	2 / 3		**4**			
